# A systematic comparison of deep learning methods for EEG time series analysis

**DOI:** 10.3389/fninf.2023.1067095

**Published:** 2023-02-23

**Authors:** Dominik Walther, Johannes Viehweg, Jens Haueisen, Patrick Mäder

**Affiliations:** ^1^Data-Intensive Systems and Visualization Group (dAI.SY), Technische Universität Ilmenau, Ilmenau, Germany; ^2^Institute of Biomedical Engineering and Informatics, Technische Universität Ilmenau, Ilmenau, Germany; ^3^Faculty of Biological Sciences, Friedrich Schiller University, Jena, Germany

**Keywords:** recurrent neural networks, feed forward neural networks, time series analysis, attention, transformer networks

## Abstract

Analyzing time series data like EEG or MEG is challenging due to noisy, high-dimensional, and patient-specific signals. Deep learning methods have been demonstrated to be superior in analyzing time series data compared to shallow learning methods which utilize handcrafted and often subjective features. Especially, recurrent deep neural networks (RNN) are considered suitable to analyze such continuous data. However, previous studies show that they are computationally expensive and difficult to train. In contrast, feed-forward networks (FFN) have previously mostly been considered in combination with hand-crafted and problem-specific feature extractions, such as short time Fourier and discrete wavelet transform. A sought-after are easily applicable methods that efficiently analyze raw data to remove the need for problem-specific adaptations. In this work, we systematically compare RNN and FFN topologies as well as advanced architectural concepts on multiple datasets with the same data preprocessing pipeline. We examine the behavior of those approaches to provide an update and guideline for researchers who deal with automated analysis of EEG time series data. To ensure that the results are meaningful, it is important to compare the presented approaches while keeping the same experimental setup, which to our knowledge was never done before. This paper is a first step toward a fairer comparison of different methodologies with EEG time series data. Our results indicate that a recurrent LSTM architecture with attention performs best on less complex tasks, while the temporal convolutional network (TCN) outperforms all the recurrent architectures on the most complex dataset yielding a 8.61% accuracy improvement. In general, we found the attention mechanism to substantially improve classification results of RNNs. Toward a light-weight and online learning-ready approach, we found extreme learning machines (ELM) to yield comparable results for the less complex tasks.

## 1. Introduction

Electroencephalography (EEG) is a non-invasive method for recording and analyzing brain activity. Given the low amplitude of the recorded signal, even an eye blink or unintentional muscle contractions create noise in the recordings, complicating the identification of a patient's mental condition. To overcome this problem, researchers traditionally focused on handcrafted feature extraction based on e.g., short-time Fourier transform (STFT) (Griffin and Lim, 1984), discrete wavelet transform (DWT) (Shensa, 1992), or tensor decomposition (Naskovska et al., 2020) to remove noise and focus on the relevant signals. Typically, the generated spectrograms are represented as images and then classified by, e.g., feed-forward networks (FFNs) (Montana and Davis, 1989). Automation of such analyses not only requires high accuracy but their embedding into usage scenarios, such as neurofeedback applications (Hammond, 2007) or brain-computer interfaces (BCI) (Schalk et al., 2004) to classify mental states also require efficient processing. However, these methods have to be calibrated manually for the image generation when specific parameters, e.g., the sampling frequency, have changed. This step requires extensive expert knowledge as otherwise important features might be neglected during preprocessing. Furthermore, these methods can be time-consuming, if the number of EEG channels increases since some of the methods propose a window and channel-wise time-frequency analysis (Tabar and Halici, 2016). Hence, previous studies often merely evaluate their methods on low channel EEG data, i.e., fewer than the clinical routine of 21 channels (Tabar and Halici, 2016; Ni et al., 2017; Mert and Celik, 2021; Yilmaz and Kose, 2021).

In the last decade, gated recurrent neural networks (RNN) like long short term memory (LSTM) (Hochreiter and Schmidhuber, 1997) and gated recurrent unit (GRU) (Chung et al., 2014) have been demonstrated to yield superior results when analyzing and classifying time series without the need for complex preprocessing and hand-crafted feature extraction. Thereby, manual configuration effort and the need for expert knowledge in signal analysis can be drastically reduced, while achieving state of the art results. In order to increase the predictive power of these approaches, they face a constant evolution with notable improvements. Such improvements include bidirectional RNN topologies and the attention mechanism that has stimulated many new network topologies beyond RNNs. More recent studies, propose time-convolving neural networks and demonstrate that they can yield high predictive performance on time series like audio signals (Oord et al., 2016; Bai et al., 2018). More specifically, Bai et al. (2018) propose a network topology based on temporal convolutions, which achieves remarkable results on popular datasets thereby outperforming LSTM and GRU topologies. In contrast to these more complex approaches, also methods based on simplified RNNs like echo state networks (ESN) achieved good (Bozhkov et al., 2016), respectively even superior results (Sun et al., 2019). As a FFN based counterpart of ESNs we reference to extreme learning machines (ELM), which were utilized for EEG classification tasks by Tan et al. (2016) and Liang et al. (2006), reaching superior results while further reducing the computational complexity.

In this paper, we systematically compare a large variety of RNN and FFN topologies as well as the influence of topological variants, e.g., bidirectional networks and attention mechanisms for EEG analysis. We do not focus on a specific medical application, but rather aim to compare the performance of each network topology based on benchmark EEG recordings. To the best of our knowledge recurrent and feed-forward topologies have never been compared on the same EEG dataset and with the same preprocessing pipeline before. We evaluate all approaches on three different EEG datasets: the well-known benchmark DEAP, a seizure detection task, and an in-house frequency entrainment dataset. Thus, we aim to answer the following research questions:

(RQ 1) *Recurrent topologies:* Which recurrent topology shows advantages for EEG time series classification in comparison between non-gated, gated, and random high dimensional mapping approaches?
(RQ 2) *Feed-Forward topologies:* Are feed-forward topologies based on convolution and self-attention suitable for EEG time series classification without further preprocessing methods?
(RQ 3) *Advanced architectural concepts:* Can extensions for LSTMs, like attention and bidirectionality, improve the performance for these networks for EEG time series classification?

Our results indicate that feed-forward networks yield advantages compared to RNNs without additional concepts. Nonetheless, applying attention to RNNs yielded notable performance increases and even surpasses feed-forward topologies for some of the investigated datasets.

The rest of the paper is organized as follows, Section 2 provides a brief summary of use cases and problems related to automated EEG analysis and introduces the step-by-step explanation of the typical workflow from the recording of the raw EEG signal to the final analysis result. Furthermore, the studied network topologies are discussed in detail. In addition, the different topological variations, like bidirectional networks and attention are explained. In this section, we will also explain the used datasets, input representation, and chosen parameters for each of the trained network architectures. In Section 3, we show different classification strategies and approaches mentioned by various publications based on the preprocessing methods and architectures used. Additionally, we discuss the different results for each of the presented topologies. Last, we discuss some limitations of our work, introduce potential future research directions and conclude on the different methods compared in this paper.

## 2. Methods

### 2.1. Applications and problems of EEG analysis

**Figure 1:**
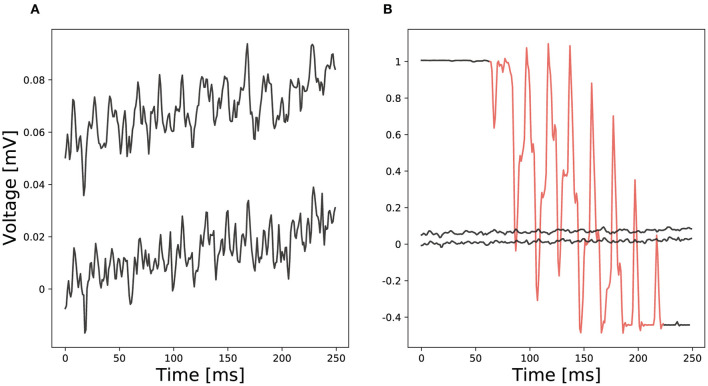
Comparison of two EEG examples: **(A)** Ordinary EEG recordings from two different electrodes and **(B)** a red marked channel with transient interferences compared to ordinary EEG recordings.

In general, analyzing EEG data is a challenging task with many difficulties (Vallabhaneni et al., 2021). Due to typically low amplitude signals in the μ*V* range (cp. Figure 1A), small interferences can distort a signal making it unusable (cp. Figure 1B red section compared to ordinary EEG recordings). We denote an interference as any part of a signal that is not directly generated by brain activity or brain activity that is not directly produced as result of an experimental stimulus. It is hard to remove interferences from a signal since these often show similar characteristics as the actual signal. To remove transient interferences before analyzing an EEG signal, various methods have been proposed, e.g., linear regression or blind source separation (Urigüen and Garcia-Zapirain, 2015). Nevertheless, none of them is supposed to work perfectly and remaining interferences may cause erroneous analysis results (Hagmann et al., 2006).

Another problem can be the placement and number of electrodes that capture brain activity. Not all regions of the brain are equally active during experiments and some regions are more dominant than others. When less electrodes are used, activation could be missed during the recording which results in no features.

To avoid such errors it is advisable to use a higher number of electrodes and to cover all areas of the head. When the number of electrodes used increases, the time and effort required to preprocess the data increases as well. This can be critical for time-frequency transforms which typically process signals channel- or window-wise (Li et al., 2016; Tabar and Halici, 2016).

In recent years, deep learning neural network approaches have been applied to a wide range of neuroscientific problems like feedback on motor imagery tasks (MI) (Tabar and Halici, 2016), emotion recognition (Ng et al., 2015), seizure detection (Thodoroff et al., 2016) and many other tasks (Gong et al., 2021) (see **Table 4**). These studies typically apply standard convolutional and recurrent neural networks (Craik et al., 2019). Many studies use handcrafted features as input for deep neural networks. However, extracting features can be time-consuming and often requires expert domain knowledge to extract features which represent the signal correctly. To avoid loss of information during the preprocessing phase, the aim of neurobiological analysis should be an analysis of raw data. If more information is provided to the neural network, better results can be expected. To the best of our knowledge, no study exists that systematically compares feed-forward and recurrent neural networks in all their flavors for raw signal EEG data analysis.

### 2.2. Automated EEG analysis workflow

**Figure 2:**
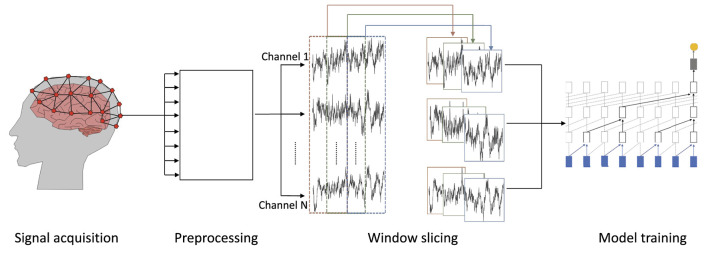
Overview of the workflow for processing EEG data: (1) signal acquisition—EEG data are recorded, (2) preprocessing—recorded data are preprocessed and noise is removed by filters, (3) window slicing—the resulting waveforms are divided into windows of equal size, which may overlap, and (4) model training—on the windowed and preprocessed wave forms.

In this subsection, we discuss the workflow for automated EEG data analysis from the recording of data to the eventual prediction (cp. Figure 2).

#### 2.2.1. Signal acquisition

We focus on EEG recordings as a non-invasive and cost efficient method to measure brain activity with electrodes placed directly on the scalp (Craik et al., 2019) (cp. Figure 2).

#### 2.2.2. Preprocessing

Preprocessing of data, such as filtering the signal and removing interferences, is an important part of training neural networks in general. Poorly preprocessed data ultimately yield poor network inference performance which can hardly be compensated by training methodology and network topology (Hagmann et al., 2006). This processing is particularly important for EEG signals which, due to their low amplitude, can be strongly altered by only small influences such as unintended muscle contractions. For this reason, almost all EEG data are bandpass filtered directly after recording to remove noise distorting the signal. An often used frequency range for EEG data analysis is 1–40 Hz. The filter range might also depend on the experimental setup during the EEG recording. Transient interference removal is another important part of preprocessing. Interferences influence a signal in a significant way and often even distort a signal such that it is nearly impossible to recognize its actual waveform (cp. Figure 1). Different methods such as linear regression or blind source separation were proposed to remove interferences. For heavily distorted signals, like shown in Figure 1 a threshold detection can track and remove the interference. After removing interferences and noise, the preprocessed data can be used as input for deep neural networks.

#### 2.2.3. Window slicing

EEG signals may contain many data points, depending on the sampling rate and duration of a recording. Often, it is not feasible to analyze a complete recording due to prohibitive compute and memory requirements which result from an excessive input length. It is, therefore, common to apply window slicing to generate data frames and to incrementally analyze these smaller snippets of a signal rather than a whole recording at once (Tabar and Halici, 2016; Gao et al., 2019). Thereby, the size of a window and a potential overlap of successive windows are hyper-parameters of the respective analysis and depend on its goal (cp. middle of Figure 2). For example, the detection of slow theta brain waves requires larger windows to capture a full wave within the window while alpha and beta brain waves can be captured in a smaller window.

#### 2.2.4. Model training

**Figure 3:**
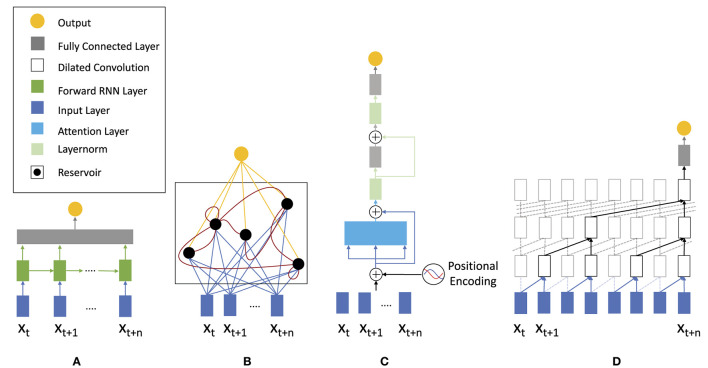
Neural network architectures applicable for analyzing time series: **(A)** traditional recurrent neural network (RNN) consisting of an input layer (blue), a forward layer (green), and a fully interconnected layer, **(B)** recurrent echo state network, **(C)** feed-forward transformer architecture utilizing attention for time series analysis, and **(D)** temporal convolutional neural network (TCN) using dilated convolutions.

The goal here is to select, parameterize, and train a suitable model architecture. Below, we discuss model topologies applicable for analyzing and specifically classifying EEG time series data (cp. Figure 3), which we then systematically evaluate on different EEG datasets in Section 2.5. Once the initial architectural choice is made, hyper-parameters are varied and optimized to improve prediction performance results. In this work we study a variety of different topolgies. These include the basic RNN as well as the most prominent recurrent networks GRU and LSTM to investigate the advantages of gated cells. As representatives for feed-forward networks we use the TCN and Transformer-Encoder topology since both of these models have shown superior results for raw time series prediction (Ingolfsson et al., 2020). Lastly, we include ESN and ELM as reservoir computing models since these are often overlooked in the literature but have shown promising results in high-dimensional time series prediction (Pandey et al., 2022; Viehweg et al., 2022).

### 2.3. Recurrent neural networks

Recurrent neural networks (RNN) (Rumelhart et al., 1988) are especially suitable to process sequential data as their topology contains feedback loops that enable the network to build up and maintain a state, sometimes referred to as memory. In contrast, a feed-forward topology (FFN) does not offer this capability and is stateless in between different inputs.

#### 2.3.1. Basic RNN

The key concept of a RNN is the cell state *c*^(*t*)^ that is connected via weight matrices in a network topology. For the basic RNN cell, the cell state *c*^(*t*)^ is calculated as:


(1) $$\begin{array}{l} c^{\left(t\right)}=tanh\left(W_{cc}c^{\left(t-1\right)}+W_{cx}x^{\left(t\right)}+b\right) \end{array}$$

where *x*^(*t*)^ is the current input, *W*_*cc*_ and *W*_*cx*_ are weight matrices, and *b* is a bias term. By incorporating state *c*^(*t*−1)^ in this calculation, the current state is influenced by the previously shown sequence.

**Figure 4:**
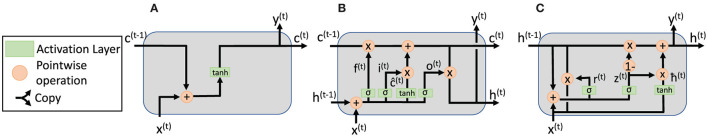
Schematic representation of non-gated and gated RNN cells: **(A)** basic RNN cell without any gates that is also representative for the reservoir of an ESN, **(B)** gated LSTM cell, and **(C)** gated GRU cell.

In theory, a basic RNN cell (cp. Figure 4A) should be capable of classifying long input sequences. However, in practice these cells suffer from vanishing and exploding gradient problems when longer sequences are processed and long-term relationships within EEG input data are relevant for signal analysis. To mitigate these problems, gated recurrent neural networks, most prominently long short-term memory (LSTM) and gated recurrent unit (GRU), have been proposed. These networks are considered among the most effective sequence modeling techniques today. While the basic RNN cell consists of a single layer with tanh activation, LSTM and GRU cells are more complex. Their key concept is different gates added to each of the states (cp. Figures 4B, C). These gates can learn what information is more or less relevant for further processing and regulate the flow of information through the network. A different approach that aims to overcome the problems of gradient descent-based learning are echo state networks (ESN) that use randomly initialized reservoir weights and merely a non-iterative learning of the output weights.

#### 2.3.2. Long short term memory

The LSTM cell (Hochreiter and Schmidhuber, 1997) consists of three gates that shall help to overcome the problem of vanishing and exploding gradients (cp. Figure 4B). The first gate within an LSTM cell is a forget gate *f*^(*t*)^ computing what information is required in the current cell state:


(2) $$\begin{array}{l} f^{\left(t\right)}=\sigma \left(W_{fh}h^{\left(t-1\right)}+W_{fx}x^{\left(t\right)}+b_{f}\right), \end{array}$$

where *W*_*fh*_ and *W*_*fx*_ are weight matrices, *b*_*f*_ is the bias, *h*^(*t*−1)^ is the previous hidden state, and *x*^(*t*)^ is the current input value. The output passes a sigmoid activation function σ bounded between 1, i.e., information is fully required, and 0, i.e., information is unnecessary. The second gate is the update gate *i*^(*t*)^. It controls how much of the current input is considered when computing the new cell state:


(3) $$\begin{array}{l} {\hat{c}}^{\left(t\right)}=tanh\left(W_{ch}h^{\left(t-1\right)}+W_{cx}x^{\left(t\right)}+b_{c}\right)\\ i^{\left(t\right)}=\sigma \left(W_{ih}h^{\left(t-1\right)}+W_{ix}x^{\left(t\right)}+b_{i}\right)\\ c^{\left(t\right)}=\left(f^{\left(t\right)}*c^{\left(t-1\right)}\right)+\left(i^{\left(t\right)}*{\hat{c}}^{\left(t\right)}\right), \end{array}$$

where ${\hat{c}}^{\left(t\right)}$ refers to the tanh activated input at time step *t*. Analogous to the forget gate, the gate uses a sigmoid function which determines the importance of the respective information as *i*^(*t*)^. The new cell state *c*^(*t*)^ then becomes the combination of the information passing through the forget and the input gate, respectively. Finally, the output gate *o*^(*t*)^ controls which information of the cell state is incorporated into the cell's current output *y*^(*t*)^ and hidden state *h*^*t*^, respectively:


(4) $$\begin{array}{l} o^{\left(t\right)}=\sigma \left(W_{oh}h^{\left(t-1\right)}+W_{ox}x^{\left(t\right)}+b_{o}\right)\\ y^{\left(t\right)}=o^{\left(t\right)}*tanh\left(c^{\left(t\right)}\right). \end{array}$$

#### 2.3.3. Gated recurrent units

The GRU cell (Chung et al., 2014) was introduced in 2014 and is a simplification of the LSTM cell. The idea is to combine forget gate and input gate into a single relevance gate *r*^(*t*)^ (cp. Figure 4C). By combining them, one weight matrix can be neglected, the cell state and hidden state are merged together, and the GRU cell is therefore supposed to be faster to train. Analogous to the LSTM cell described above, the state of the relevance gate *r*^(*t*)^, the state of the updated gate *z*^(*t*)^, and the hidden state *h*^(*t*)^ are computed as follows:

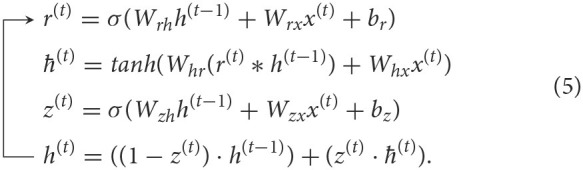



**Figure 5:**
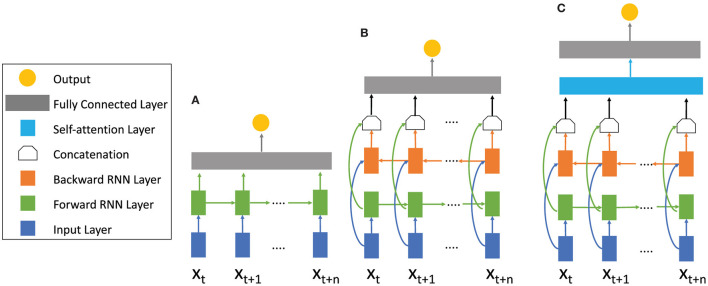
RNN architectures with different mechanisms. **(A)** Basic RNN architecture consisting of the input layer (blue) the RNN forward layer (green), and a fully connected layer (gray). The sequence is only calculated forward in time. **(B)** Bidirectional RNN architecture. In addition to the forward RNN layer a backward RNN (orange) layer is added. Information from the future and the past is calculated simultaneously and concatenated, summed, multiplied or averaged afterwards. **(C)** Attention Bidirectional RNN architecture. After the forward and backward calculations have been concatenated, an attention layer (blue) is used to pay attention to important sections of the sequence. For different RNN topologies like LSTM and GRU the cells within the forward and backward layer differ (see Figure 4).

With the help of gates, GRU and LSTM (cp. Figure 5A) are supposed to be able to analyze longer sequences without being affected by vanishing gradients. Both variations are very popular for analyzing sequential data. While GRUs are more cost efficient due to fewer parameters, the LSTM contains more training capacity but requires more computational power and longer training time.

#### 2.3.4. Echo state networks

An alternative approach to potentially overcome the problems of gradient descent-based training is the non-iteratively trained echo state networks (ESN) (Jaeger, 2001). ESNs are a prominent RNN architecture that realize the reservoir computing paradigm (Verstraeten et al., 2007). An ESN consists of three core layers: the input layer, the reservoir layer, and the output layer. Only the weights of the output layer are trained. All other weights are typically randomly initialized from a uniform distribution, i.e., those of the input layer $W_{hx}\in {\mathbb{R}}^{N^{res}\times N^{in}}$ and those of the reservoir layer $W_{hh}\in {\mathbb{R}}^{N^{res}\times N^{res}}$. A reservoir layer can be considered as a simplified RNN cell without most of the trainable parameters (cp. Figure 4A) and is denoted as:


(6) $$\begin{array}{l} h^{\left(t\right)}=\gamma \cdot h^{\left(t-1\right)}+\left(1-\gamma \right)f\left(W_{hx}x^{\left(t\right)}+W_{hh}h^{\left(t-1\right)}\right), \end{array}$$

where *x*^(*t*)^ ∈ ℝ^*N*^*in*^^ is the input, *h*^(*t*−1)^ ∈ ℝ^*N*^*res*^^ is the previous cell state, *f*(·) is an activation function, typically tanh, and γ is the leakage rate that determines how much of the ESN's previous hidden states is added to compute the new hidden state *h*^(*t*)^. During the learning phase, a single training sequence *S*_*T*_ with length T is utilized to compute the respective hidden states {*h*^(*i*)^, …, *h*^(*i*+*T*)^}. The learning phase of an ESN is separated in two steps. First an initialization phase is done whereby the states {*h*^(0)^, …, *h*^(*i*−1)^} are discarded, but the activation for each respective neuron is initialized (Jaeger, 2001). This process is often referred to as the washout phase (Malik et al., 2016). Second is the training phase, where the previous hidden states are added to the current hidden states, in relation to the leakage rate γ. The resulting matrix *H* ∈ ℝ^*N*^*res*^×*T*^, which is based on the hidden states, is then mapped to the expected outputs *Y* ∈ ℝ^*N*^*out*^×*T*^ via a linear regression with $y^{\left(t\right)}=W_{yh}\cdot h^{\left(t\right)}$ according to:


(7) $$\begin{array}{l} W_{yh}=YH{\left(HH^{T}+\beta I_{N^{r}}\right)}^{-1}, \end{array}$$

with β as regularization coefficient and $I_{N^{r}}$ as unity matrix. For classification tasks, we train a reservoir for each class *c* within the dataset. We call this an ensemble of predictors, where each predictor processes the input file, and the class is chosen based on the predictor with the smallest error. For evaluation, each sample is processed by each predictor and is assigned to the class with the lowest prediction error (Forney et al., 2015).

#### 2.3.5. Bidirectional architecture

In some applications, it can be helpful to process a sequence's previous as well as future information simultaneously. That is the concept of a bidirectional RNN combining two RNN layers, one for processing input data in a forward manner and one for processing input data in a reverse manner (Schuster and Paliwal, 1997) (cp. Figure 5B). The outputs of both layers are concatenated and eventually processed by a fully connected layer. This architectural approach is applicable for any RNN cell and has often been demonstrated to improve network performance when processing complex sequences in general (Huang et al., 2015; Yin et al., 2017) and to analyze EEG data (Ni et al., 2017; Chen et al., 2019). Ogawa et al. (2018) found that a bidirectional architecture improves accuracy in comparison to a basic RNN model by 1.1% for video classification based on the user's favors.

#### 2.3.6. Attention

The attention mechanism is an imitation of human behavior. Rather than considering the entire previous input when computing the next output, a network learns which previously computed hidden states are beneficial to compute an output for a given new input. This approach is also applicable to any RNN cell and even to feed-forward networks as we will discuss in the next subsection. Attention computes the relation between the current input *x*^(*t*)^ and previous inputs {*x*^(1)^, …, *x*^(*t*−1)^} represented as hidden states {*h*^(1)^, …, *h*^(*t*−1)^} with the help of an attention layer (Bahdanau et al., 2014; Cheng et al., 2016) (cp. Figure 5C):


$$\begin{array}{l} a_{i}^{\left(t\right)}=v^{T}tanh\left(W_{h}h_{i}+W_{x}x^{\left(t\right)}+W_{\tilde{h}}{\tilde{h}}^{\left(t-1\right)}\right)\\ s_{i}^{\left(t\right)}=\frac{exp\left(a_{i}^{t}\right)}{\sum_{i^{\prime }=1}^{n}exp\left(a_{i^{\prime }}^{t}\right)}. \end{array}$$

The attention calculation results in a distribution of probabilities of the previous values. With the probability distribution $s_{i}^{t}$, an adaptive summary vector can be calculated. Cheng et al. (2016) proposes to replace the previous hidden state *h*^(*t*−1)^ used in Equations (2)–(4) by a cell and hidden memory tape ${\tilde{c}}^{\left(t\right)}$ and ${\tilde{h}}^{\left(t\right)}$:


$$\begin{array}{l} {\tilde{h}}^{\left(t\right)}=\overset{t-1}{\underset{i=1}{\sum }}s_{i}^{t}\cdot h_{i}\\ {\tilde{c}}^{\left(t\right)}=\overset{t-1}{\underset{i=1}{\sum }}s_{i}^{t}\cdot c_{i}. \end{array}$$

The cell and hidden memory tape contain all the previous cell and hidden states {*c*^(1)^, …, *c*^(*t*−1)^} and {*h*^(1)^, …, *h*^(*t*−1)^}, respectively. Attention allows the network to give certain previous hidden states more weight in generating the current output than others. Thereby, rather than utilizing a single hidden state *h*^(*t*−1)^ the network gains access to all previously processed hidden states and can weigh their importance.

### 2.4. Feed-forward networks

In contrast to recurrent neural networks, feed-forward networks like multilayer perceptrons (MLPs) and convolutional neural networks (CNNs) do not have any feedback connections between the output of a neuron and its input, i.e., input information *x* passes a series of operations and only influences the network's current output *y*. Traditional feed-forward networks were therefore not well suited to analyze time series data. Due to their non-recurrent nature, temporal dependencies could not be modeled well and extending the input size toward longer sequences became prohibitively expansive due to an exponentially growing number of parameters. However, there are more recent architectural concepts to overcome these limitations of FFNs in sequence processing, while preserving their benefits over RNNs, i.e., parallelizable training and being less prone to vanishing and exploding gradients. Below, we discuss three fundamental approaches for applying feed-forward architectures to time series data classification.

#### 2.4.1. Transformer

**Figure 6:**
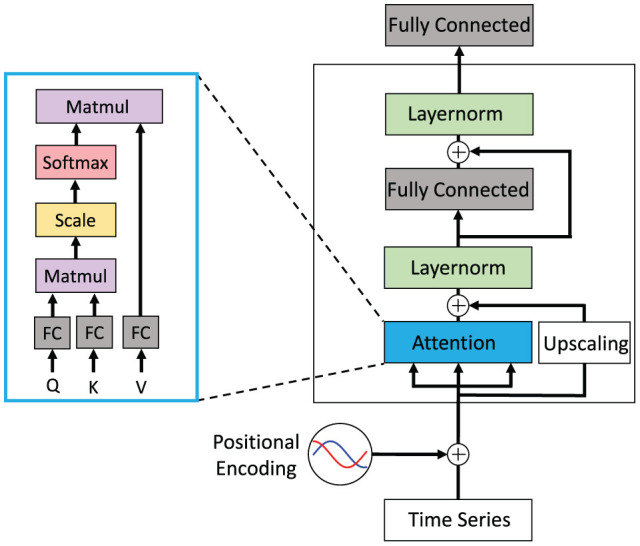
Conceptual view of a Transformer block that shows a single attention layer that is typically realized as multiple attention heads. This Transformer block may be stacked multiple times and may be arranged in an encoder-decoder architecture with attention layers spanning across encoder and decoder. Figure replicated from Vaswani et al. (2017).

The feed-forward Transformer architecture makes extensive use of the attention concept. It has been demonstrated to achieve superior results especially in the field of natural language processing (NLP) in recent years (Vaswani et al., 2017). Each block of the Transformer consists of an attention layer, a fully connected layer, and a final classification layer. Residual connections are added around the attention and fully connected layer followed by a layer normalization (cp. Figure 6). The attention mechanism is implemented as a multiplication of the input with three different weight matrices *W*_*Qx*_, *W*_*Kx*_, *W*_*Vx*_ and computed as:


(8) $$\begin{array}{l} \alpha \left(Q,K,V\right)=s\left(\frac{Q\cdot K^{T}}{\sqrt{d_{k}}}\right)V, \end{array}$$

with Q, K, and V as Query, Key, and Value, respectively. The scaling factor is denoted as *d*_*k*_ and the Softmax function as *s*(·). For solving NLP problems, such as machine translation, the Transformer typically follows an encoder-decoder structure (Vaswani et al., 2017). For classification problems only the encoder without the decoder part is used since only a single output conveying the classification result is required. Therefore, the model will be referred to as Transformer-Encoder in the rest of the paper.

#### 2.4.2. Temporal convolutional network

**Figure 7:**
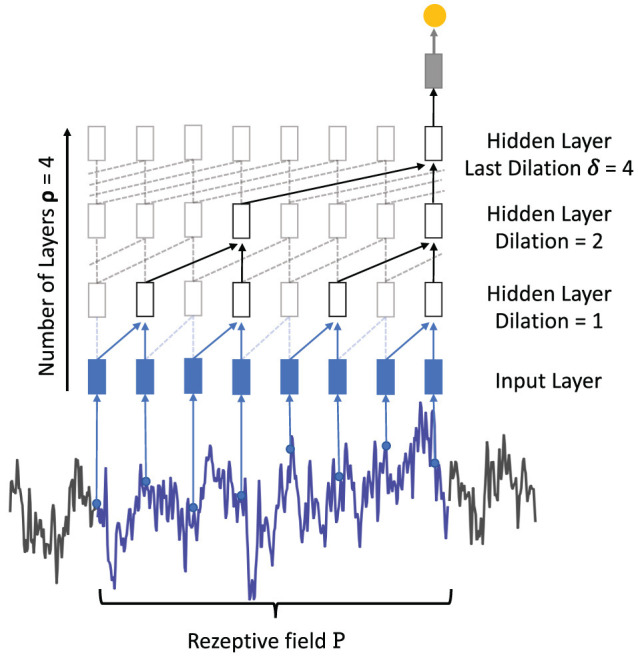
Visualization of a single TCN block χ for time series classification. Each time step of the time series is taken as input (for better visibility some points are removed from the example). With a filter size λ = 2, two samples are convolved, respectively. In the following hidden layers, the dilation ∈ [1,2,4] with a maximum dilation δ = 4 skips several samples and increases the length of the receptive field.

An alternative feed-forward architecture for the analysis of sequential data is the temporal 1D convolutional network (TCN) that is based on two key concepts (Bai et al., 2018). First, causal convolutions keep the temporal relationship between inputs, i.e., the input at time *x*^*t*^ can only be convolved with an input of *x*^*t*−*n*^. Second, since a fully convolutional architecture would exponentially grow in depth with an increasing input length, dilated convolutions (Oord et al., 2016; Bai et al., 2018) are proposed and filter over larger input windows with a defined number of input are being skipped. Figure 7 illustrates the dilated convolutions concept where the first hidden layer convolves each two successive input values while the second hidden layer convolves two inputs but skips the intermediate one. The dilation rate δ_*i*_ increases exponentially with each hidden layer added to the network, starting with a dilation rate of 1. The number of TCN layers can therefore be derived by calculating the logarithm of the maximum dilation rate *log*_2_(*d*_*i*_*max*__). Due to the dilation concept, TCNs are theoretically able to process sequences of any length without facing the problem of vanishing or exploding gradients. The amount of dilation per convolutional layer influences the receptive field P of a network calculated as:


(9) $$\begin{array}{l} \text{P}=1+\left(\lambda -1\right)\cdot \chi \cdot \underset{i}{\sum }\delta_{i}, \end{array}$$

where χ is the number of TCN blocks, λ is the filter length, and δ_*i*_ is the dilation rate of the respective hidden layer. The example in Figure 7 consists of one TCN block, the last dilation is denoted as 4 and the filter size was set to 2. Using Equation (9) for dilated convolutions results in a receptive field of length 8. Without the use of dilated convolutions, the length of the receptive field would be 5 with the same amount of parameters. The TCN has been evaluated against LSTM and GRU on common sequence modeling datasets and demonstrated comparable and often better performance across the various tasks (Bai et al., 2018).

#### 2.4.3. Extreme learning machines

Huang et al. (2004) proposed the extreme learning machine (ELM) in which an input of lower dimensionality is mapped into a high dimensional state space via a random mapping. The random mapping is defined as $W_{hx}\in {\mathbb{R}}^{N^{res}\times N^{in}+1}$ and $W_{hx}\sim U\left(-0.5,0.5\right)$ with U as uniform distribution and *N*^*in*^, *N*^*res*^ ∈ ℕ being the dimensionality of the input and the reservoir, respectively. With these mappings, the hidden state *h*^(*t*)^ at time *t* is calculated as:


(10) $$\begin{array}{l} h^{\left(t\right)}=f\left(W_{hx}x^{\left(t\right)}\right), \end{array}$$

with *x*^(*t*)^ as the input at time step *t* and *f*(·) as the activation function. These mappings are collected for *T* ∈ ℕ time steps and then mapped to the correct output by calculating the weights of the outputs *W*_*yh*_. Within the scope of this work, we view the data as a time-series to predict. We use the approach of Forney et al. (2015), to learn $W_{yh}^{c}$ for each class *c* and predict the time series of the validation dataset to classify by the lowest predictive error.

### 2.5. Experimental setup

We studied the four RNN topologies introduced above, i.e., the basic RNN, the GRU, the LSTM, and the ESN. Additionally, we studied them in a bidirectional architecture and added the attention concept. We also studied the three FFN topologies introduced above, i.e., the Transformer-Encoder, the TCN, and the ELM. Each of the network topologies are evaluated for intra-subject classification tasks.

#### 2.5.1. Datasets

We utilize three datasets to comparatively evaluate the introduced methods. Two of those are known benchmarks in the field of EEG analysis: the seizure and the DEAP dataset. Furthermore, we added the much larger frequency entrainment dataset since the feature learning effectiveness of deep neural networks heavily depends on large training sets. We describe the datasets as used in this study and based on the raw data, generated from the mentioned measurements. In cases of frequency cut offs done during the measurement, we report them but do not use any additional statistics to imprint specific features into the dataset that were not found by the neural networks themselves. All datasets are available within the reported frequency ranges and are not preprocessed any further. The filtering is oftentimes done during the measurement procedure and can be part of the recording process.

##### 2.5.1.1. Seizure dataset

The seizure dataset includes five different classes (Tzallas et al., 2009). Each class contains 100 single-channel EEG recordings. Classes Z and O have been recorded from five healthy participants with eyes opened and closed, respectively. Classes F and N are measured at different brain regions, with F being recorded at the epileptogenic zone and N being recorded at the hippocampal formation, both without any seizures. Class S contains recordings of actual seizures. We define three classification tasks of increasing complexity for the seizure dataset, i.e., Task 1: S-Z, Task 2: S-N-Z, and Task 3: S-N-O-F-Z, that have been studied before and therefore allow for comparison with previous work (Tzallas et al., 2009).

##### 2.5.1.2. DEAP dataset

The DEAP dataset is a public emotion recognition dataset where 32 participants watched 40 1-min-long music videos while their neural activity was recorded with a 32-channel EEG cap (Koelstra et al., 2011). The electrodes were placed according to the 10–20 system. After watching the video, each participant was asked to rate the strength of their emotions on a Likert scale from 1 to 9 according to four classes: arousal, dominance, liking, and valence. Analogous to earlier studies, we derive four binary classification problems, one per emotion, distinguishing between a low <5 and a high ≥5 emotion rating.

##### 2.5.1.3. Frequency entrainment dataset

Salchow et al. (2016) published a study with 12 participants stimulated by 20 different flickering light frequencies. The flickering light was intended to investigate the individual resonance and entrainment effects of the participants. Given the different intrinsic brain oscillations of the participants leading to different resonance and entrainment effects when using fixed stimulation frequency for all participants, the actual stimulation frequency per participant was chosen relatively to her or his individual alpha frequency (α). The alpha frequency was measured before the actual experiment. Each stimulation frequency was shown to a participant a total of 30 times with 40 light flashes. Brain activity was recorded using a 124-channel EEG. The data were recorded at a sampling rate of 1 kHz and then filtered between 2 and 30 Hz using a zerophase Butterworth filter (Salchow et al., 2016) since the resonance and entrainment phenomena are expected in this frequency range and anything else is considered noise. The task for this dataset is to classify the respective light frequency a participant was exposed to based on the recorded EEG data. The task is especially challenging since a trained classifier needs to distinguish between almost identical frequencies, e.g., 0.50 × α and 0.55 × α. Moreover, the different frequencies stimulate almost the same brain regions. For higher frequencies above 1.30 × α, Salchow et al. (2016) describe that participants notice the flash as a continuous light instead of a flickering light, which makes it hard to distinguish between.

#### 2.5.2. Preprocessing

Our evaluation differs from previous studies that often used customized and dataset-specific features for classification, such as Chen et al. (2019) and Du et al. (2020). However, in this study we mainly focus on papers that also evaluate their method on windowed signals. We argue that this approach, albeit possibly yielding worse accuracy, reflects a more realistic scenario of analyzing raw time series signals as model input. Therefore, we trained all networks on raw EEG recordings that were solely bandpass filtered to the frequency ranges reported in Table 1 to remove frequencies unrelated to neural activity of interest (cp. Section 2.2). We removed the distorted channels 42 and 63 from the frequency entrainment dataset by comparing maximum signals across all channels and selecting those that strongly deviated from the average maximum. We assume that the problem arose from an electrode failure and was present for all participants.

**Table 1:** Overview of the utilized datasets.

	**Seizure**	**DEAP**	**Frequency entrainment**
Sampling frequency [Hz]	173.61	128.00	1,000.00
Recorded EEG channels	1	32	122^*^
Number of classes	2, 3, 5	2	20
Bandpass filtering range [Hz]	0.53–40	4–45	2–30
Train:test split [%]	80:20^***^	80:20^**^	70:30^**^
Participants	–	32	9
Total training samples [tsd.]^****^	4.0, 5.9, 9.8	1.7	110.0
Chance level [%]	50, 33, 20	58, 64	11

* We removed two channels of the originally 124 recorded due to an electrode problem.

** We used the recordings from a single participant for an initial hyper-parameter search with a 70:20:10 train, test, validation split.

*** The best fitting hyper-parameter set was estimated for Task 2 with a 70:20:10 train, test, validation split.

**** Training samples are measured per participant, the seizure dataset does not provide a participant wise splitting.

#### 2.5.3. Hyper-parameter tuning and training

We used the Kotila (2019) grid-search package to identify the most suitable hyper-parameters per dataset. More specifically, we used the recordings of one participant in a 70:20:10 train, test, validation split to perform this search for the RNNs, the TCN, and the Transformer-Encoder. We do not expect that the hyper-parameters differ substantially when tuning them for another participant, because of a similar data distribution. The standard deviation, mean, maximum, and minimum values across all the participants are in similar ranges and the recording procedure as well as the task does not change across participants. Since the seizure dataset provides only one dataset and is not divided per participant, the hyper-parameter search was done for the 3 class classification problem with the same split mentioned above. We searched for an optimal setting of window length, window step size, batch size, learning rate, momentum, learning rate decay, dropout ratio, network depth, hidden size, dilation rate (TCN only), scaling factor, and number of heads (Transformer-Encoder only). We utilized grid search as hyper-parameter optimization strategy. The upper and lower boundaries for each hyper-parameter are shown in Table 2. Additionally, we optimized the hyper-parameters for the ELM and the ESN based on a set of up to 100 randomly seeded weight matrices. Thereby, we searched for the most suitable parameterization of hidden size *N*^*r*^, leakage rate γ, regularization coefficient β, density of the weight matrix *d*(*W*_*hh*_) (ESN only), and spectral radius ρ (ESN only). Table 3 shows the discovered hyper-parameters per dataset.

**Table 2:** Hyper-parameter boundaries for Talos grid-search.

**Parameter**	**Lower boundary**	**Upper boundary**
Window length	32	1,024
Window step size	1	512
Batch size	8	256
Learning rate	0.001	0.1
Momentum	Disabled	Enabled
Learning rate decay	Disabled	Enabled
Dropout ratio	0	0.7
Network depth	1	3
Hidden size	1	1,024
Dilation rate	8	64
Scaling factor	0.1	10
Number of heads	1	4
Leakage rate	0	1
Regularization coefficient	10^−13^	10^2^
Density of the weight matrix	0	1
Spectral radius	0	1.5

**Table 3:** Hyper-parameter selection for the utilized datasets.

**Hyper-parameter**	**Seizure**	**DEAP**	**Frequency entrainment**
Window length	1,024	1,024	256
Window step size	128	128	32
**(Gated) RNN, TCN, and Transformer-Encoder**			
Batch size	64	16	64
Learning rate	0.1	0.001	0.001
Momentum	–	0.9	0.8
Learning rate decay	Yes	No	Yes
Optimizer^*^	SGD/AdamW	Adam/AdamW	SGD/AdamW
Dropout ratio	0.4	0.5	0.4
Network depth	1	1	1
Hidden size^****^	64	32	128
Number of heads^*****^	1	1	1
Loss function	Cross-entropy	Cross-entropy	Cross-entropy
δ^**^	16	32	16
*d*_*k*_ ^***^	1.0	1.0	1.0
**ESN and ELM**			
*N* ^*r*^	10	50/250	1000
γ	0/1	1	0
β^******^	9.60·10^−13^, 3.89	1·10^−4^, 100	1·10^−4^
*d*(*W*^*hh*^)	0.5	0.5	0.5
ρ	0.995	0.995	0.995

* AdamW is applicable to the Transformer-Encoder.

** Dilation rate is applicable to the TCN.

*** Scaling factor is applicable to the Transformer-Encoder.

**** Hidden size for the Transformer-Encoder corresponds to the feed-forward network.

***** Number of heads only applicable for the Transformer-Encoder.

****** Different optima for Arousal, Valence as well as Seizure 2, 3 and 5 and between ESN and ELM given as maximum and minimum found optimal value.

The TCN and all recurrent networks except ESN were trained using the Keras framework on Tesla V100 GPUs with the SGD or Adam as optimizers. The Transformer-Encoder was implemented with PyTorch. For the Transformer-Encoder, we used the AdamW optimizer, as this kind of network requires a different learning strategy than the other presented networks (Popel and Bojar, 2018). We noticed that the recurrent architectures suffered from bad network initialization multiple times and did not improve during training. This was especially the case for the DEAP dataset and, thus, the training had to be restarted. We did not observe this behavior during the training for the Transformer-Encoder and the TCN. This phenomenon is mentioned by other studies that describe a similar behavior as a characteristic of training RNNs (Sutskever, 2013). That is why we explain the poor training behavior by the nature of RNNs rather than the chosen hyper-parameters based on one specific participant.

To compare the classification capabilities of each of the presented architectures, we used the accuracy metric. We applied early stopping during each training with a patience of 50 epochs to stop the training if the model does not improve anymore.

## 3. Evaluation

### 3.1. Status Quo in EEG classification with deep learning

Automatic EEG time series analysis has gained an increasing interest in recent years due to the success of deep learning in a wide range of tasks (Gong et al., 2021). Various studies focused on EEG classification and have proposed interesting approaches to tackle the problem (cp. Table 4). Before transforming the recorded signals, all considered primary studies applied filtering methods to remove noise and restrict the analysis to relevant frequency ranges. The most commonly used filter technique is bandpass filters. Various different preprocessing methods like discrete wavelet transform (DWT) and differential entropy (DE) have been proposed to extract representations like different frequency bands from raw EEG signals. However, the most common signal representations are time series followed by selected frequency bands. Table 4 shows CNNs and LSTMs as the most prominently studied model topologies. Yang et al. (2020) proposed a bidirectional LSTM for EEG classification tasks. They found that bidirectional architectures perform better for EEG analysis than LSTMs without this design. The attention mechanism has also been studied in combination with the LSTM topology to solve such tasks (Zhang et al., 2019; Du et al., 2020). Both publications report that the attention mechanism improves results by about 6–7% compared to LSTM architectures without attention. A popular approach is to combine two topologies, like CNN and LSTM. In this combination, the CNN is used as a feature extractor that delivers the input to the LSTM which classifies based on these features. Cai et al. (2018), Isuru Niroshana et al. (2019), and Jeong et al. (2019) found that RNNs can benefit when a CNN is applied as features extractor. But Cai et al.'s results also indicate that the combined architecture reduces the accuracy for some subjects. The most prominent datasets used by the authors include DEAP (Koelstra et al., 2011), BCI competition IV (BCIIV, 2008), PhysioNet (PhysMi, 2009), and SEED (SeedBci, 2013). Other publications evaluate their approaches on proprietary datasets representing, e.g., MI tasks (Lu et al., 2017; Tang et al., 2017; Cai et al., 2018) and emotion recognition (Choi and Kim, 2018; Keelawat et al., 2019). However, it is hard to compare the performance of the proposed methods even for the same dataset due to often varying experimental protocols like choosing specific EEG channels or reducing the number of classes to distinguish between (Dose et al., 2018; Zhang et al., 2019). This often leads to better performing models due to the removed channels and classes which are hard to distinguish. The best accuracy on the DEAP dataset was achieved by a TCN architecture with 72.9% (Yang and Liu, 2019) for classification with windowed signals. Many reviews of deep learning methods for EEG time series classification have been published (Craik et al., 2019; Gong et al., 2021; Vallabhaneni et al., 2021). However, none of them compare the reviewed methods with respect to the same experimental setup. We argue that a systematic comparison of the proposed as well as other deep learning methods is required to evaluate their potential for EEG analysis and yield guidelines for data scientists and researchers in this area.

**Table 4:** Previously studied deep learning EEG analysis methods grouped by dataset.

**References**	**EEG Ch**.	**Preprocessing**	**Signal representation**	**Model topology**	**Classes**	**Accuracy (%)**
**DEAP** (Koelstra et al., 2011)						
Chen et al. (2019)	32	FFT	PSD features	Attention BiGRU	2	67.2
Yang and Liu (2019)	32	DE	PSD features	TCN	2	72.9
**BCI competition IV** (BCIIV, 2008)						
Amin et al. (2020)	22	Raw	Time series	1D-CNN	4	74.8
**Physionet MI dataset** (PhysMi, 2009)						
Dose et al. (2018)	64	Raw	Time series	1D-CNN	2/3/4	86.49/79.25/68.51
Zhang et al. (2019)	27	TFF	Frequency bands + info	Attention LSTM	2	83.2
**SEED** (SeedBci, 2013)						
Yang et al. (2020)	62	Raw	Time series	BiLSTM	4	84.2
Du et al. (2020)	62	DE	Frequency bands	Attention LSTM	3	91.1
**CMEED**						
Du et al. (2020)	30	DE	Frequency bands	Attention LSTM	2	91.5
**– Proprietary –**						
Cai et al. (2018)	16	Raw	Time series	1D-CNN + GRU	2	93.5
Keelawat et al. (2019)	12	Raw	Time series	CNN	2	73
Gao et al. (2019)	30	Raw	Time series	1D-CNN	2	90.0
Shamwell et al. (2016)	64	Raw	Time series	1D-CNN	2	AUC 0.72
Jeong et al. (2019)	30	Raw	Time series	1D-CNN + LSTM	5	69.5
Kaushik et al. (2018)	14	DWT	Frequency bands	BiLSTM	6, 2	93.7, 97.5
Isuru Niroshana et al. (2019)	6	Raw	Time series	1D-CNN + GRU	4	87.7
Bozhkov et al. (2016)	21	Raw	Time series	ESN+SVM	2	98.1
Sun et al. (2019)	5	Raw	Time series	FE-ESN	5	98.33

Model topologies aggregated by “+” indicate an approach combining both topologies. DE, differential entropy; DWT, discrete wavelet transform; TFF, time-frequency feature extraction; FFT, fast fourier transform; PSD, power spectral density.

### 3.2. Comparative evaluation

Table 5 shows our measured classification performance on the test set of the three studied datasets (rows) for the seven network topologies introduced in Section 2 (columns). We ordered datasets and their tasks with increasing complexity from top to bottom in Table 5. Since the frequency entertainment dataset is unbalanced due to the different stimulation frequencies (cp. Section 2.5), we included the F1-score for each model. The following paragraphs discuss our results and observations with regards to the research questions stated in Section 2.5.

**Table 5:** Accuracy (%) of different neural network topologies for multiple EEG datasets.

	**Recurrent networks**				**Feed-forward networks**		
**Dataset**	**Basic RNN**	**LSTM**	**GRU**	**ESN**	**Transformer**	**TCN**	**ELM**
**Seizure**							
Task 1: S-Z	93.16	**100.00**	**100.00**	**100.00**	**100.00**	**100.00**	81.58
Task 2: S-N-Z	72.28	97.56	97.72	34.48	80.17	**98.27**	44.83
Task 3: S-N-O-F-Z	58.20	63.52	68.88	44.90	58.41	**90.37**	45.92
**DEAP**							
Emotion class: arousal	65.23	67.78	68.97	68.75	66.12	65.36	**70.31**
Emotion class: valence	61.42	68.06	**68.40**	53.12	63.09	62.01	64.06
**Frequency entrainment**							
Accuracy	56.65	68.32	73.72	14.44	77.90	**82.33**	17.72
F1-Score	59.05	68.67	73.90	12.60	73.53	**82.46**	15.98

The best performing architectures for a specific task are marked in bold.

We observe widely varying classification accuracies across the different network topologies per dataset and task. In general, we observe a better performance of feed-forward topologies compared to recurrent topologies across most of the studied classification tasks (cp. Table 5). Recurrent as well as feed-forward topologies benefit from more advanced architectural concepts like gates in the LSTM and GRU topologies, attention in the Transformer-Encoder topology, or convolution in the TCN topology. These more advanced topologies achieve superior performance compared to less complex topologies, i.e., the basic RNN, the ESN, and the ELM. Furthermore, the more advanced topologies suffer less from a decreasing performance with growing input dimensionality, i.e., the number of analyzed channels, and problem complexity, i.e., the number of predicted classes. However, the advanced topologies performed better during the training and oftentimes achieved 95% and higher training accuracy values, but could not generalize well on the test set. This behavior indicates that these models overfitted. Nonetheless, reducing the model size and depth reduced the overfitting problem, but also led to lower validation performance. When comparing the model parameters as shown in Table 6, we notice that larger models performed overall better in comparison to smaller ones. Nonetheless, when searching for the best possible set of hyper-parameters (cp. Table 2), even models larger than the ones reported in this work did not yield better results. Thus, we argue that the number of trainable parameters is not directly related to the overall performance of the model.

**Table 6:** Model parameters for each architecture and dataset in [tsd.].

**Network topology**	**Seizure**	**DEAP**	**Frequency entrainment**
Basic RNN	4.3, 4.4, 4.5	2.2	35.2
LSTM	17.2, 17.3, 17.4	8.6	132
GRU	12.9, 13, 13.1	6.5	99
ESN	0.01	1.4	137
Transformer-Encoder	152, 217, 346	74	738
TCN	672	105	1,396
ELM	0.01	2.7/9.2	22.3
Attention LSTM	152, 217, 349	76	793
Bidirectional LSTM	34.4, 34.6, 34.9	17.2	263
Attention + bidirectional LSTM	304, 435, 697	152	1,585

#### 3.2.1. RQ1: Recurrent topologies

A direct comparison of all recurrent networks shows that the basic RNN and the ESN yield the lowest accuracy across the different datasets and tasks. The basic RNN cell does not achieve results comparable to the other presented methods on seizure Task 1. Furthermore, the basic RNN shows a notable performance reduction with an increasing number of classes for the seizure tasks as well as worse performance on the other, higher dimensional datasets. Similar to LSTM and GRU, the ESN achieves 100% accuracy on the least complex seizure Task 1. However, we observe substantial performance deficits for all the other tasks, with an overall lower accuracy than the basic RNN. We expected the ESN to perform better than the basic RNN since Chattopadhyay et al. (2019) and Vlachas et al. (2020) have shown that the ESN is comparable with the LSTM and GRU on time series prediction. This was not the case for any of the evaluated datasets. We argue that the ESN's non-iterative learning approach is not sufficient to learn important features to distinguish between more similar classes. For the gated recurrent networks, we observe that the GRU and LSTM consistently outperform the basic RNN as well as the ESN across all classification tasks demonstrating that their advanced control of information flow allows them to better adapt to high dimensional EEG time series. When comparing GRU and LSTM, we observe a better performance for the GRU across all datasets. For the DEAP as well as the frequency entrainment datatset we tested whether the differences between both cells are significant by applying a statistical *t*-test. However, the results are not significantly different when comparing both cells directly. As already stated by some studies, GRU and LSTM perform similar and it is more important to find the best working parameter set than choosing the architecture (Chung et al., 2014). Nevertheless, we consider GRU superior compared to LSTM due to the lower number of model parameters (cp. Table 6).

#### 3.2.2. RQ2: Feed-forward topologies

Overall, feed-forward topologies yield better performance than recurrent topologies. We observe similar performance trends for the ELM comparable to the basic RNN and the ESN. The ELM cannot compete with self-attention and convolutional approaches and performs substantially worse on the other investigated tasks. Surprisingly the ELM achieved the best performance for the DEAP arousal task. However, since the ELM follows a comparable training process as the ESN, we argue that the full batch learning approach is not suitable for high-dimensional hard to distinguish EEG recordings as the results show for our frequency entrainment dataset.

The recently proposed Transformer-Encoder is designed to take advantage of large amounts of data with the dataset used in Vaswani et al. (2017) being distinctly larger than the training datasets used in our study. While for the Task 1 and 2 of the seizure dataset the Transformer-Encoder performed well compared to other approaches, its accuracy notably drops for Task 3 with five classes to differentiate. We argue that the complexity of the third task, paired with the relatively low amount of training data resulted in the low accuracy. We observe a similar behavior for the DEAP dataset. However, the results from our frequency entertainment dataset demonstrate the true potential of Transformer-Encoder networks. Though having a higher input dimensionality in terms of analyzed EEG channels, the Transformer-Encoder is capable to outperform the previously discussed topologies yielding a 4.18% higher accuracy than the GRU architecture and achieving the second-best result across all topologies. We hypothesize that the results of the Transformer-Encoder can be further improved when having sufficient and rich training samples. The TCN, as another feed-forward approach, yields a rather constant performance across all investigated datasets. It achieves the highest accuracy across all studied topologies for most of the tasks. As observed for the other architectures, the accuracy of the TCN decreases with increasing problem complexity. This behavior is demonstrated by the achieved accuracies for the different seizure tasks. Based on the results for the seizure dataset, we argue that the TCN is capable of extracting features even on a small number of training samples and can overcome the limitations of the Transformer-Encoder topology which requires a large number of training samples. For the DEAP dataset, we observe that the TCN and Transformer-Encoder had problems to distinguish between high and low emotion classes and stayed almost at guessing for the DEAP emotion task. We hypothesize, that the information about the emotion is present in frequency ranges the TCN may cannot recognize well. In contrast, one specific property of recurrent architectures is, that they usually forget important information laying far in the past. This property makes RNNs sensible to higher frequency ranges and one can argue that emotions are recognizable in higher frequency ranges. Zheng and Lu (2015) confirms this finding.

#### 3.2.3. RQ3: Advanced architectural concepts

We extended the previously trained LSTM with an attention mechanism, used it in a bidirectional setup, and studied both extensions simultaneously. Table 7 reports results of these experiments. For all tested datasets, attention yielded an increase in accuracy with the largest being an 24.75% increase for seizure Task 3. This is comparable to the TCN for this task and achieves the best results for the seizure Task 2. For the DEAP and the frequency entrainment datasets, we also observe significant accuracy improvements compared to the LSTM without attention. Some previous studies report a slight performance improvement when the LSTM cell is used in a bidirectional setup (Ni et al., 2017). In contrast, we observed a 0.01–2.81% degraded performance across all datasets except seizure Task 2 when applying this architecture. The benefit of the bidirectional setup heavily depends on the task and we argue that a 'look-ahead' may be highly beneficial for sequence to sequence tasks like machine translation but is of less help when predicting a class based on a full sequence. The combination of attention and bidirectional setup yields an improved performance across most of the investigated datasets. However, for all seizure tasks as well as the frequency entrainment datatset, the performance is lower than that observed for the attention mechanism alone. Surprisingly, for the DEAP task, the combination of attention and bidirectional setup yielded an increased performance. We hypothesize that the combination of both, attention and bidirectionality can be beneficial for some EEG classification tasks. However, the doubled number of weights due to the bidirectional LSTM (cp. Table 6) can negatively impact the model performance and shows only minor improvements compared to the model only utilizing attention.

**Table 7:** Accuracy (%) for different LSTM variations compared to the TCN.

**Dataset**	**LSTM variations**							**TCN**
	**–**	**Attention**		**Bidirectional**		**Attent.+ bidirect**.		
**Seizure**								
Task 1: S-Z	100.00	100.00		100.00		100.00		100.00
Task 2: S-N-Z	97.56	**98.57**	(+1.01)	98.14	(+0.58)	95.55	(-2.01)	98.27
Task 3: S-N-O-F-Z	63.52	88.27	(+24.75)	60.71	(-2.81)	86.22	(+22.70)	**90.37**
**DEAP**								
Emotion class: arousal	67.78	76.64^*a*^	(+8.86)	68.77	(-0.01)	**77.26** ^*a*^	(+9.48)	65.36
Emotion class: valence	68.06	77.07^*a*^	(+9.01)	67.97	(-0.09)	**77.43** ^*a*^	(+9.37)	62.01
**Frequency entrainment**								
Accuracy	68.32	69.84	(+1.52)	68.09	(–0.23)	69.78	(+1.44)	**82.33**
F1-Score	68.67	69.97	(+1.30)	68.85	(+0.18)	69.98	(+1.31)	**82.46**

The colored numbers indicate the difference in comparison to an LSTM cell without the different applied mechanisms. Lower case letter (a) next to the reported value indicates significant differences between LSTM without mechanisms and the different variations. The best performing architectures for a specific task are marked in bold.

### 3.3. Limitations

Determining the best-performing model configuration via hyper-parameter tuning is typically an expensive and time-consuming activity. We tuned the hyper-parameters for the RNNs, TCN, and Transformer-Encoder as described in Section 2.5, but did not perform an additional optimization for the RNNs with attention and the bidirectional setup. Therefore, it is possible that some of the parameters still could be optimized and improved. However, we do not expect a substantial change in the results and argue that we only compared for differences among the topologies rather than absolute accuracy. Other studies mentioned handcrafted feature extraction methods for EEG time series analysis. We did not further investigate time-consuming and subjective methods to extract the best possible features. Therefore, it might be possible to achieve better absolute performance with such specifically tailored feature extraction methods.

Based on Transformers, a multitude of extensions of the approach were proposed in recent years, e.g., Dai et al. (2019) and Zhou et al. (2021), which circumvent problems regarding the memory usage and the length of the input. With both of these approaches being designed for time-series prediction and in comparison small training size we do not expect an improvement in using these advancements of Transformers. We reiterate our assumption that the Transformer could achieve better results with more training data.

Given the low amplitude of the EEG signal, the recordings are prone to noise. Depending on the strength of the noise it is possible that it could have a negative impact on the topologies. We did not insert additional noise or remove parts of the signals to test the robustness of each model. Lim et al. (2021) shows that the accuracy of RNN topologies can drop when a strong noise is added to the dataset. Zanghieri et al. (2019) and Zhang and Wu (2019) indicate that FFNs are not that much influenced when noise is added to the signal. However, we do not expect that other EEG recordings differ much from the ones presented in our study. All investigated datasets are not further preprocessed to remove the noise recorded during the experiments.

### 3.4. Future research

The proposed methods are still among the best performing topologies for deep learning tasks. However, there are other interesting architectural concepts which are not investigated in this work. These are especially brain-inspired intelligence approaches such as spiking neural networks (Tavanaei et al., 2019). Lately published studies such as SAM (Yang et al., 2022a), Spike-Based Continual Meta-Learning (Yang et al., 2022c), or ensemble models (Yang et al., 2022b) are promising methods to solve neuroscientific problems.

As previously mentioned, EEG time series prediction has many difficulties (Vallabhaneni et al., 2021). Recently published learning and regularization strategies have shown to improve the learning process of the neural networks presented in this work. Such strategies can be Hamilton-Jacobi-Bellman equations (Reddy et al., 2018), Curiculum Learning (Teutsch and Mäder, 2022), or Synaptic Scaling (Hofmann and Mäder, 2021). These learning approaches could help to reduce the overfitting which was observable during our experiments and could be further investigated.

Lastly, these models could be compared with respect to other metrics such as robustness with erroneous EEG signal recordings which are sometimes overlooked during the preprocessing.

## 4. Conclusions

In this paper, we trained ten different state-of-the-art neural network model topologies and methods and compared their results on the popular seizure dataset, the emotion dataset DEAP as well as the larger frequency entrainment dataset. More specifically, we compared models' classification performance on deep recurrent architectures for time series classification including GRUs, LSTMs, and ESNs as well as on feed-forward architectures like Transformer-Encoders, TCN, and ELMs. The experimental results indicate that the TCN yields better performance for EEG time series data compared to RNNs and is less dependent on a high number of training examples, which are required for the Transformer-Encoder. In general, all feed-forward architectures were easier to train. As described in Section 3, networks with recurrence suffered from bad initialization which led to no learning progress. This behavior was not observed for feed-forward networks. We argue, that our results justify the use of feed-forward topologies like TCN and the Transformer-Encoder in contrast to previous standard topologies which utilize recurrence or high dimensional random mappings (RQ1 and RQ2). Furthermore, we investigated the influence of bidirectional and attention mechanisms as these were previously proposed by individual studies. We found that the attention mechanism increases the LSTM's performance for all studied datasets and achieved even better results than the TCN in some experiments. In contrast, the bidirectional mechanism had a negative impact on our results and the LSTM cell did not benefit from calculating the sequence forward and backwards in time. We also noticed that the combination of both mechanisms does not always improve the model performance but requires more memory since the model parameters are doubled due to the bidirectionality. Thus, we do not recommend applying bidirectional mechanism to RNNs for EEG time series classification (RQ3). We evaluated all architectures on raw signals without handcrafted feature extraction for all the datasets. Our results show that it is possible to solve different tasks without major adjustments to the training pipeline. However, for all presented datasets we had to deal with the overfitting problem and could not reach the best performance on the DEAP dataset, compared to other methods that use hand-crafted features for classification.

## Data availability statement

The data analyzed in this study is subject to the following licenses/restrictions: The DEAP [1] and the seizure [2] datasets are publicly available from their original authors. The frequency entrainment dataset has been recorded according to a protocol that was not GDPR compliant and therefore German legal regulations prevent us from publicly sharing this dataset. That is the recorded data potentially contains identifying or sensitive patient information that has not been authorized by the respective participant for public sharing. However, we support justified validation requests on this dataset, e.g., by executing validation code on our side. Such requests shall be directed to Vice President for Research of Technical University of Ilmenau (vpf@tu-ilmenau.de) as the responsible person for that dataset. [1] https://www.eecs.qmul.ac.uk/mmv/datasets/deap/; [2] http://web.archive.org/web/20070812162213/http://www.epileptologie-bonn.de/front_content.php?idcat=193.

## Author contributions

DW worked on conceptualization, formal analysis, methodology, validation, visualizaton, and writing. JV contributed to formal analysis, methodology, validation, and writing. JH contributed to data curation, review, and editing. PM contributed to conceptualization, writing, review, and editing. All authors contributed to the article and approved the submitted version.
